# Biochemical Characterization of Thermostable Acrylamide Amidohydrolase from *Aspergillus fumigatus* with Potential Activity for Acrylamide Degradation in Various Food Products

**DOI:** 10.1007/s00284-023-03544-1

**Published:** 2023-12-05

**Authors:** Ashraf S. A. El-Sayed, Hala N. Elghamry, Marwa A. Yassin

**Affiliations:** Enzymology and Fungal Biotechnology Lab, Department of Botany and Microbiology, Faculty of Science, Zagazig University, Zagazig, 44519 Egypt

## Abstract

Acrylamide is the major by-product of the Maillard reactions in foods with the overheating processes of L-asparagine-rich foods with reducing sugars that usually allied with neurotoxicity and carcinogenicity. Several approaches have been used to prevent the formation of acrylamide, however, degrading the already formed acrylamide in foods remains unequivocal. Acrylamide hydrolyzing enzyme “amidohydrolase” is one of the most promising enzymes for acrylamide degradation in foods. So, amidohydrolase “amidase” from thermotolerant *Aspergillus fumigatus* EFBL was purified to their electrophoretic homogeneity by gel-filtration and ion-exchange chromatography, with overall purification folds 2.8 and yield 9.43%. The apparent molecular subunit structure of the purified *A. fumigatus* amidase was 50 kDa, with highest activity at reaction temperature of 40 °C and pH of 7.5 The enzyme displayed a significant thermal stability as revealed from the value of *T*_*1/2*_ (13.37 h), and thermal denaturation rate (*K*_*r*_ 0.832 × 10^–3^ min) at 50 °C, with metalloproteinic identity. The purified enzyme had a significant activity for acrylamide degradation in various food products such as meat, cookies, potato chips, and bread as revealed from the HPLC analysis and LC–MS analysis. So, with the purified amidase, the acrylamide in the food products was degraded by about 95% to acrylic acid, ensuring the possibility of using this enzyme in abolishing the toxic acrylamide in the foods products. This is the first report exploring the potency of *A. fumigatus* amidase for an actual degradation of acrylamide in foods efficiently. Further biochemical analyses are ongoing to assess the affinity of this enzyme for selective hydrolyses of acrylamide in foods, without affecting the beneficial stereochemical related compounds.

**Supplementary Information:**

The online version contains supplementary material available at 10.1007/s00284-023-03544-1.

## Introduction

Acrylamide is one of the major by-products of Maillard reactions in foods during the processing, due to the reactivity of L-asparagine with the reducing sugars by overheating at 120 °C for at least 25 min [1]. The higher amounts of acrylamide was present in foods rich with proteins and carbohydrates, such as meat products, crisp bread and French fries (4 mg/kg) [2]. The consequences of acrylamide exposure have been allied to neurotoxicity and carcinogenicity [3], thus acrylamide was classified by the World Health Organization (WHO) and International Agency for Research on Cancer as a Group 2A [4, 5]. Due to the high contents of L-asparagine and reducing sugars, French fries are the most vulnerable foods to acrylamide formation, confirming the role of overheating on provoking the acrylamide formation [6]. The acrylamide carcinogenicity derives from their adduct formation with hemoglobin [7], as well as, the by-products of acrylamide degradation “epoxy glycidamide” has a higher reactivity to form DNA adducts (glycidamide-DNA), producing hazardous cellular metabolic effects [3, 8]. Foods rich with reducing carbohydrates “glucose, fructose,” and L-asparagine were reported to have a higher amounts of acrylamide with the cooking temperature [9]. The admissible dose of acrylamide/day should be less than 10 μg/kg body weight [10]. The toxicity of acrylamide monomer elaborates from its low molecular weight and high water solubility that easily passing via the cellular biological membranes [11], with subsequent ability to undergo various metabolic transformation by reacting with numerous sub-cellular targets [3, 9, 11, 12]. The unique chemical structure of acrylamide by having α and β-unsaturated carbonyl groups “Michael-type” facilitates its reactivity with thiols of glutathione, forming mercapturic acid that urinary excreted as the common detoxification way [5, 7, 9, 11, 13–15]. Also, acrylamide can undergo oxidative transformation by cytochrome P450, producing a glycidamide derivative with higher reactivity for DNA and proteins than the parent acrylamide [4, 5, 7, 14, 16]. Thus, the conjugation of acrylamide with GSH results in depletion of cellular GSH pool, changing the redox states of the cell that can potentially affect gene expression directly [11]. Acrylamide can be found in human placenta and breast milk, easily transferable to fetus and to the newborn infants [5, 8, 17]. Thus, several approaches were hypothesized to reduce the risk of higher accumulation of acrylamide in foods: 1- Pre-acrylamide formation process, preventing the acrylamide formation by L-asparaginase and 2- Post-acrylamide formation process, degradation of the acrylamide present in food. Abolishing of acrylamide formation upon using microbial L-asparaginase has been frequently studied [18]; however, the microbial degradation of acrylamide in foods and identity of acrylamide-degrading enzyme has received less attention. Microbial degradation of acrylamide in foods is one of the most sophisticated approaches for alleviating the hazardous effect of extra amounts of acrylamide in human body [19].

Acrylamide amidases (Acrylamidase, amidohydrolase, EC 3.5.1.4) catalyzes the acrylamide hydrolysis to acrylic acid and ammonia [20] and was partially characterized from various bacterial species, *Ralstonia eutropha*, *Bacillus licheniformis, Enterobacter aerogenes, Stenotrophomonas acidaminiphila,* and *Moraxella osloensis* [21–24], and fungal isolates *Aspergillus* oryzae [15]. However, the lower catalytic and conformational stability and possibility of enzyme usage for degradation of acrylamide in food products are the challenge, for further technological applications. With the massive implementation of fungi in different fermentation processes in food industries for production of enzymes, organic acids, and flavors [22], the ability of fungi for degrading acrylamide in food products remains equivocal. Thus, the objective of the current work was to assess the potentiality of fungi for acrylamide degradation, to purify the acrylamide amidase, and to evaluate their ability of acrylamide degradation in food products.

## Materials and Methods

### Screening for Acrylamide-Degrading Fungi

Twenty-five fungal isolates from our laboratory stock [23][24–28] were grown on modified Potato Dextrose Agar (PDA) media (250-g potato extract, 2-g acrylamide, and 20-g agar–agar dissolved in 1 L distilled water) and incubated for 8 days at 30 °C. Among the experimented fungi, four fungal isolates mainly *Aspergillus fumigatus*, *A. flavus*, *A. niger,* and *A. awomari* had a reliable visual growth on the acrylamide containing PDA media. The isolates *Aspergillus fumigatus, A. flavus,* and *A. niger* were originally recovered from the soil samples from the Botanical Garden of Faculty of Science, Zagazig University, Zagazig, Egypt, in March/2021. Different acrylamide concentrations (0.01, 0.5, and 1.0%) were amended to the modified PDA media, the fungal culture was incubated at 30 °C for 10 days, and the fungal growth was observed, as well as modified Potato Dextrose Broth (PDB) media (250 g potato extract/ liter distilled water) were prepared, amended with different concentrations of acrylamide (0.01, 0.5 and 1.0%) and then inoculated with a plug of 7-day-old fungal culture, per 250-ml Erlenmeyer conical flaks. The cultures were incubated for 10 days at 30 °C, and the fungal biomass was collected, washed, and then assessed [29].

### Morphological and Molecular Identification of the Potent Acrylamide-Degrading Fungi

The experimental fungal isolates were identified based on their macroscopic and microscopic features by growing on PDA and Czapek-Dox media, according to the reference keys [30–32]. The most potent fungal isolate-degrading acrylamide was further molecularly confirmed based on its internal transcribed spacer (ITS) sequence [33–35]. The fungal mycelia (~ 0.2 g) were pulverized in liquid nitrogen and dispensed in 1-ml CTAB extraction buffer (2% CTAB, 2% PVP40, 0.2% 2-mercaptoethanol, 20-mM EDTA, 1.4-M NaCl in 100-mM Tris − HCl, pH 8.0). The genomic fungal DNA was used as template for PCR, with the primer sets ITS4 5′-GGAAGTAAAAGTCGTAACAAGG-3′ and ITS5 5′-TCCTCCGCTTATTGATATGC-3′. The reaction mixture contains 10-μl 2 × PCR master mixture (Cat. No. 25027), 1-μl gDNA, 1 μl of each primer (10 pmol/μl), and 20 μl distilled water. The PCR conditions were programmed as follows: initial denaturation 94 °C for 4 min, denaturation at 94 °C for 30 s, annealing at 53 °C for 20 s, extension at 72 °C for 40 s for 35 cycles, and final extension at 72 °C for 5 min. The amplicons were analyzed by 1.5% agarose gel in 1 × TBE buffer (Cat# AM9864) with 1-kb DNA ladder (Cat. #PG010-55DI). The amplicon was sequenced by Applied Biosystems, HiSQV Bases with the same primers. The sequence was annotated by non-redundant BLAST search on NCBI database, aligned with Clustal W and the phylogenetic tree was constructed with neighbor-joining method of MEGA X [36].

### Acrylamide Amidase Activity and Protein Concentrations Assay

The most potent acrylamide-decomposing fungal isolate was grown on potato dextrose broth medium with 0.5% acrylamide for 10 days at 30 °C, then the fungal biomass was collected by filtration, and then intracellular crude proteins were extracted [18, 37–39]. Briefly, ten grams of the fungal biomass were pulverized in liquid nitrogen and then dispensed in 50-ml potassium phosphate buffer (pH 7.0) with 1-mM dithiothreitol and 1-mM EDTA. The mixture was vortexed for 5 min, centrifuged at 8000 rpm for 10 min at 4 °C, and the supernatant was used as a crude source for acrylamide amidase. The activity of acrylamide amidase was determined by Nessler’s reagent [19]. The reaction mixture contains 50-mM acrylamide in 20-mM potassium phosphate buffer (pH 7.0) and 500 μl of enzyme preparation in 1-ml total volume, the reaction was incubated at 40 °C for 15 min, stopped by adding 10% Trichloroacetic acid, and then the supernatant was amended with Nessler’s reagent (ADWIC, N0178111) [38, 39]. Blanks of the crude enzyme extract without acrylamide and substrate without enzyme, amended with Nessler’s reagent, were used. The developed color was measured at λ_425_ nm, and the concentration of ammonia was calculated from the inference of authentic concentrations of ammonium sulfate [19, 24, 25]. The activity of amidase (1 unit) was expressed by the amount of enzyme releasing 1 μmol of ammonia per mg protein under the standard conditions. The enzyme protein concentration was measured by Folin’s reagent [40], regarding to bovine serum albumin as authentic one. The activities and concentrations of L-asparaginase were assessed.

### Purification and Molecular Subunit Structure of *Aspergillus fumigatus* Acrylamide Amidase

*Aspergillus fumigatus* was grown on potato dextrose broth medium amended with 0.5% acrylamide, incubated at the desired conditions, the fungal pellets were collected, and then washed by potassium phosphate buffer. The fungal biomass was pulverized in liquid nitrogen and the intracellular crude proteins were extracted [18, 38, 39, 41]. The crude enzyme was fractionally concentrated with 20-kDa cut-off dialyzer (Cat.# 546–00051), against polyethylene glycol 6000 [24, 26–28, 37, 38] followed by 30-kDa Ultracentrifuge membrane (Amicon, Millipore) at 10,000 rpm for 15 min at 4 °C. The enzyme was further purified by gel-filtration with Sephadex G200 column [33–35, 39]. The activity of amidase was measured by the standard assay, and the most active fractions were collected, concentrated, and their homogeneity was checked by denaturing PAGE [42]. The most active fractions were collected, further concentrated by 30-kDa Ultracentrifuge membrane, and further purified by ion-exchange chromatography with DEAE-Sepharose column [36–38]. The most active amidase and molecularly homogeneous fractions were selected. The molecular homogeneity and subunit structure of the purified acrylamide amidase were checked by SDS-PAGE [42], normalizing to authentic protein marker (Puregene, Cat. #PG-PMT2962, 315–10 kDa).

### Biochemical Properties of the *A. fumigatus* Acrylamide Amidase

The biochemical properties of the purified *A. fumigatus* amidase such as reaction temperature, reaction pH, and thermal stability were investigated [19]. The reaction mixture containing 50-mM acrylamide dissolved in 20-mM Tris–HCl (pH 8–9), potassium phosphate buffer (5–7), and citrate phosphate buffer (4–7) was incubated at 40 °C for 15 min and then the enzyme activity was measured, as mentioned above. The standard reaction mixture was incubated at various temperatures (10 to 60 °C) and then the enzymatic activity was measured as mentioned above. The thermal stability of the purified amidase was assessed by pre-incubating the enzyme at 4, 20, 30, 40, and 50 °C for 30, 60, and 120 min and then measuring the residual enzyme activity as described above [18, 37, 43–45]. The impact of different cations on enzyme activity was determined by pre-incubating the apo-enzyme with different inhibitors (Ba^2+^, Fe^3+^, Ca^2+^, Hg^2+^, Fe^3+^, Al^3+^, Zn^2+^, Na^+^, Cu^2+^) for 2 h at 4 °C at final concentration 1 mM and then assessing the residual enzymatic activity. Different amino acid analogues such as 5,5′-dithio-bis-(2-nitrobenzoic acid) (DTNB), hydroxylamine, guanidine thiocyanate, iodoacetate, 3-methyl-2-benzo-thiazolinone hydrazone (MBTH), and phenylmethylsulfonyl fluoride (PMSF) were incubated with the enzyme at 1 mM for 2 h at 4 °C and then measured their residual enzyme activity.

### Food Applications of the Purified *A. fumigatus* Amidase in Different Food Products

The functionality of amidase to degrade acrylamide in different food products mainly meat, bread, cookies, and potato chips was determined [14]. One gram of the tested food products was soaked in 5 ml of the amidase preparations (85 μmol/mg/min) for 60 min at 40 °C, using distilled water as negative control. The food products were vigorously homogenized, and the homogenate was centrifuged at 8000 for 10 min and the amount of acrylamide was determined by HPLC (YOUNG In, Chromass, Korea) of reverse phase C18 column (Cat.# 959,963–902). The mobile phase was methanol/acetonitrile/water (90:5:5, v/v/v) at a flow rate 1 ml/min for 25 min, and the absorbance of acrylamide was measured at λ_203_ nm [14], compared to the authentic one (Cat. #. 79–06-1). The purity and concentration of the acrylamide in samples were determined from the retention time and peak area, normalizing to authentic one at λ_203_.

### LC–MS Analysis of Acrylamide and its Derivatives in Food Products

The prepared homogenates of the food products were defatted with hexane, evaporated, and the concentration of acrylamide and its degradation by-products were determined [18]. The acrylamide and acrylic acid concentrations were determined using LC–MS (Waters Corp., Milford, MA01757, USA). The ESI–MS-positive ion acquisition mode was carried out on a XEVO TQD triple-quadruple instrument, with column ACQUITY UPLC-BEH C18 (1.7 µm, 2.1 × 50 mm), with solvent system of acetonitrile (A) and 0.1% formic acid (B). The elution was carried out at 25 °C with a flow of 0.2 ml/min, using the following gradients: at the beginning (10% A and 90% B); up to 10 min (increase of solvent A to 90% and decrease of solvent B to 10%); from 10 to 15 min (90% A and 10% B); and from 15 min to the end (10% A and 90%). The chemical identity of the acrylamide and acrylic acids was determined reliant of their mass spectra and retention time referencing to NIST and WILEY libraries.

### Fungal Deposition

*Aspergillus fumigatus* EFBL has been deposited to the GenBank with accession # MW737636.1 and at Assiut University Mycological Center, Egypt, with deposition # AUMC14078.

### Statistical Analysis

The experiments were performed in triplicates, and the results were expressed by mean ± STDV. The statistical analysis was assessed using one-way ANOVA (analysis of variance, SPSS software v.18) test, and the means were compared with Duncan’s test at 0.05 level.

## Results

### Screening and Identification of the Potent Acrylamide-Degrading Fungi

Twenty-five fungal isolates were preliminary screened for acrylamide degradation by growing on modified potato dextrose agar with 0.2% acrylamide. A visual fluctuation was observed on the ability of the tested fungi to grow on acrylamide-containing PDA media (Data not shown). Among the experimented fungi, four isolates, namely *Aspergillus fumigatus* EFBL (MW737636.1), *A. flavus* (MT951414.1), *A. niger,* and *A. awomari* exhibited the highest reliable growth on acrylamide-containing PDA media (Fig. S1). The morphological features, conidial pigmentation, and biomass of the tested fungi in response to the different concentrations of acrylamide were assessed. From the fungal growth, a visual reduction to the fungal growth with the acrylamide concentration has been observed, in a concentration-dependent manner, with no observed growth at 0.5% acrylamide. Obviously, the growth of *A. flavus*, *A. awomari,* and *A. niger* were strongly reduced with the higher concentration of acrylamide (0.5%), unlike the noticeable tolerance of acrylamide toxicity displayed by *A. fumigatus.* At 0.5% acrylamide, the growth of *A. flavus*, *A. awomari,* and *A. niger* was reduced by about 80–90%, comparing to acrylamide-free media. However, a noticeable tolerance to acrylamide toxicity has been observed by *A. fumigatus*, as revealed from their visual growth and biomass, and at 0.5% acrylamide the fungal growth was reduced by about 5% comparing to control. Remarkably, the deep blue-colored conidial pigmentation of *A. fumigatus* EFBL was subsequently faded with the higher acrylamide concentration. The morphological feature of the potent acrylamide-hydrolyzing fungal isolate “*Aspergillus fumigatus*” is shown in Fig. S2. The morphological identity of the *A. fumigatus* was confirmed from the ITS sequence that has been deposited on GenBank with accession number MW737636.1. The tolerance of *A. fumigatus* EFBL to acrylamide reveals their possessing to powerful detoxifying system of acrylamide-degrading enzymes “amidases” that counteract the acrylamide toxicity by converting it to acrylic acid and ammonia.

### Purification, Molecular Subunit Structure, and Biochemical Properties of *A. fumigatus* Amidase

**Fig. 1:**
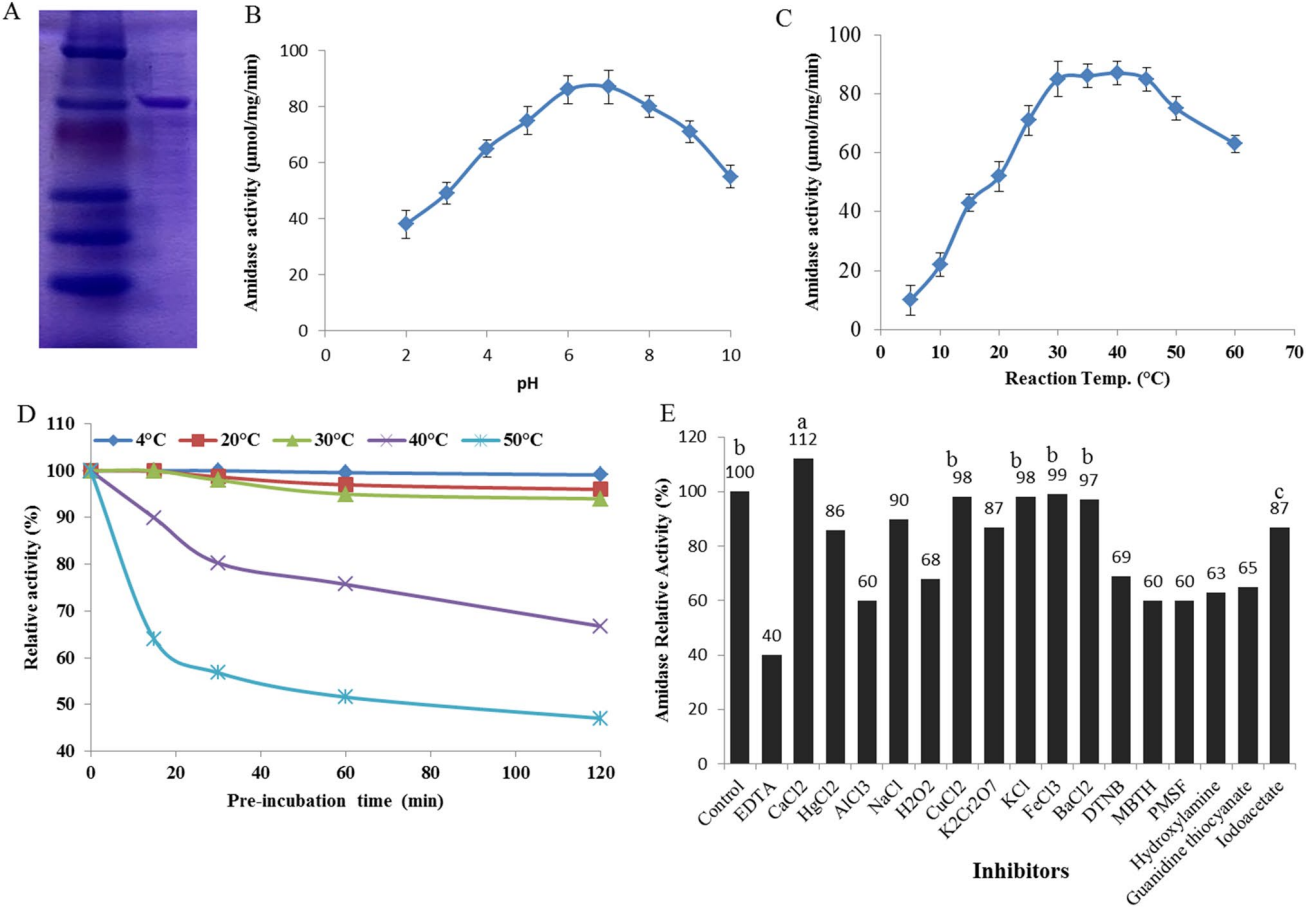
Purification of acrylamide amidase from *A. fumigatus.* The intracellular proteins from *A. fumigatus* was extracted and the enzyme was purified by Gel-filtration with Sephadex G200 column and Ion-exchange chromatography. **a** SDS-PAGE of the purified amidase from *A. fumigatus.* The effect of reaction pH (**b**), areaction temperature (**c**), as well as thermal stability (**d**) and effect of cations on the activity of the purified enzyme (**e**) was shown. The Statistical significance was assessed using one-way ANOVA test and the means were compared with Duncan’s test at 0.05 level. Accordingly, mean values with different small letters are statistically different (*p* ≤ 0.05)

*Aspergillus fumigatus* was grown on the PDB medium containing acrylamide (0.5%) for 8 days at 30 °C, then the fungal pellets were collected, washed and their intracellular proteins were extracted, and further purified by ion-exchange and gel-filtration chromatography. The overall purification profile of amidase from *A. fumigatus* EFBL is shown in Table 1. The activity of amidase was increased by 2.4 folds (73.2 μmol/mg/min), with recovery 16.6%, upon gel-filtration chromatography. The active fractions were collected, their molecular homogeneities were checked, gathered, and purified by DEAE-Sepharose column, and the enzyme was purified by 2.8 folds (85.7 μmol/mg/min) with yield 9.43%, compared to the crude enzyme (31.5 μmol/ mg/min). The active homogeneous fractions of amidase were collected, concentrated, and then checked for its molecular subunit structure by SDS-PAGE. The purified amidase shows a single band of molecular mass 50 kDa under SDS-PAGE (Fig. 1A).

**Table 1:** Purification profile of acrylamide amidase from *A. fumigatus*

Step	Total activity (U)	Total protein (mg)	Specific activity (μmol/mg/min)	Recovery (%)	Purification fold
Crude Enzyme	3611	114.6	31.5	100	1
Ultracentrifuge membrane	1525.8	42	36.4	42.3	1.2
Sephadex G200	599.8	8.2	73.2	16.6	2.4
DEAE-Sepharose	340.5	4	85.2	9.43	2.8

The effect of reaction temperature on the activity of purified *A. fumigatus* amidase was assessed by incubating the mixture at 10, 20, 30, 40, 50, and 60 °C and then measuring the enzyme activity by the standard assay. A gradual increase in the activity of *A. fumigatus* amidase was observed with the temperature, till maximum activity at 30–40 °C, with a noticeable reduction in the enzyme activity by about 20%, comparing to control (35 °C) (Fig. 1). The conformational thermal stability of amidase in response to the pre-incubation at 4, 20, 30, 40, and 50 °C, at different incubation times was measured by the standard assay. From the thermal stability profile, the activity of amidase was reduced with the pre-incubation temperature as revealed from the thermal denaturation. The thermal kinetic parameters of the purified amidase are listed in Table 2. The half-life time (*T*_*1/2*_) of the purified *A. fumigatus* amidase was 250, 75.7, 29.9, and 16.3 h, at 4, 20, 30, and 40 °C, respectively, while the thermal denaturation rate (*Kr*) of amidase was 0.002 × 10^–3^, 0.15 × 10^–3^, 0.245 × 10^–3^, and 0.499 × 10^–3^ min, at 4, 20, 30, and 40 °C, respectively. Obviously, the thermal structural denaturation rate of the enzyme was increased with the higher temperature and with pre-incubation time. The influence of reaction pH (2–10) on the activity of purified *A. fumigatus* amidase was assessed. A remarkable increase in the activity of amidase with the reaction pH, till maximum activity at pH 7.0, is followed by a decrease in the enzyme activity with higher pHs (pH 10) (Fig. 1). A significant reduction in the activity of the enzyme was observed at highly acidic pHs (2.0–4.0) and alkaline pHs (8.5–10). Similar biochemical properties were reported for the purified amidase from *Rhodococcus* sp. [19].

**Table 2:** Thermal kinetic parameters of the purified *A. fumigatus* Amidase

Temp (°C)	T_1/2_ (h)		*Kr* (min) × 10^–3^
4	250	0.002	
20	75.7	0.15	
30	29.93	0.245	
40	16.30	0.499	
50	13.37	0.832	

^*^Half-life time (*T*_*1/2*_) was expressed by the time at which the enzyme retains 50% of its initial activity by preheating without substrate at each temperature degree

^**^Thermal denaturation rate (*K*_*r*_) was expressed by the logarithmic decreasing of enzyme activity with the time at each temperature

(ln (At/A0) =−*Kr*, where *A0* and *At* are the specific activity of Asnase at zero and *t* time

It is described by the first-order kinetic model

The effect of various inhibitors on the catalytic identity of purified amidase was assessed. The enzyme was demetallized by dialysis against 50-mM Tris–HCl of 1.0-mM EDTA, to resolve the apo-enzyme [37, 46]. The activity of the apo-enzyme was about 40% of the holo-enzyme, confirming the enzyme metalloproteinic identity. The activity of amidase was restored upon addition Ca^2+^, Cu^2+^, and Ba^2+^, followed by monovalent cations K^+^ and Na^+^ at 1 mM (Table 3). The loss of enzyme activity by demetallization with EDTA authenticates the enzyme metalloproteinic identity. The catalytic domains of the amidase were mapped from the amino acid suicide analogues, such as hydroxylamine, iodoacetate, guanidine thiocyanate, DTNB, MBTH, H_2_O_2_, and PMSF. The activity of amidase was reduced by about 30% in response to addition of PMSF, MBTH, and DTN and ensured the involvement of surface thiols amino acids on the enzyme active sites.

**Table 3:** Relative activity of purified *A. fumigatus* amidase in response to different inhibitors

Compound	Relative activity
Control	100
EDTA	40
CaCl_2_	112
HgCl_2_	86
AlCl_3_	60
NaCl	90
H_2_O_2_	68
CuCl_2_	98
K_2_Cr_2_O_7_	87
KCl	98
FeCl_3_	69
BaCl_2_	97
5,5-Dithiobis (2-nitro benzoic)	69
MBTH	60
PMSF	60
Hydroxylamine	63
Guanidine thiocyanate	65
Iodoacetate	87

### Applications of *A. fumigatus* Amidase in Degradation of Acrylamide in Food Products

**Fig. 2:**
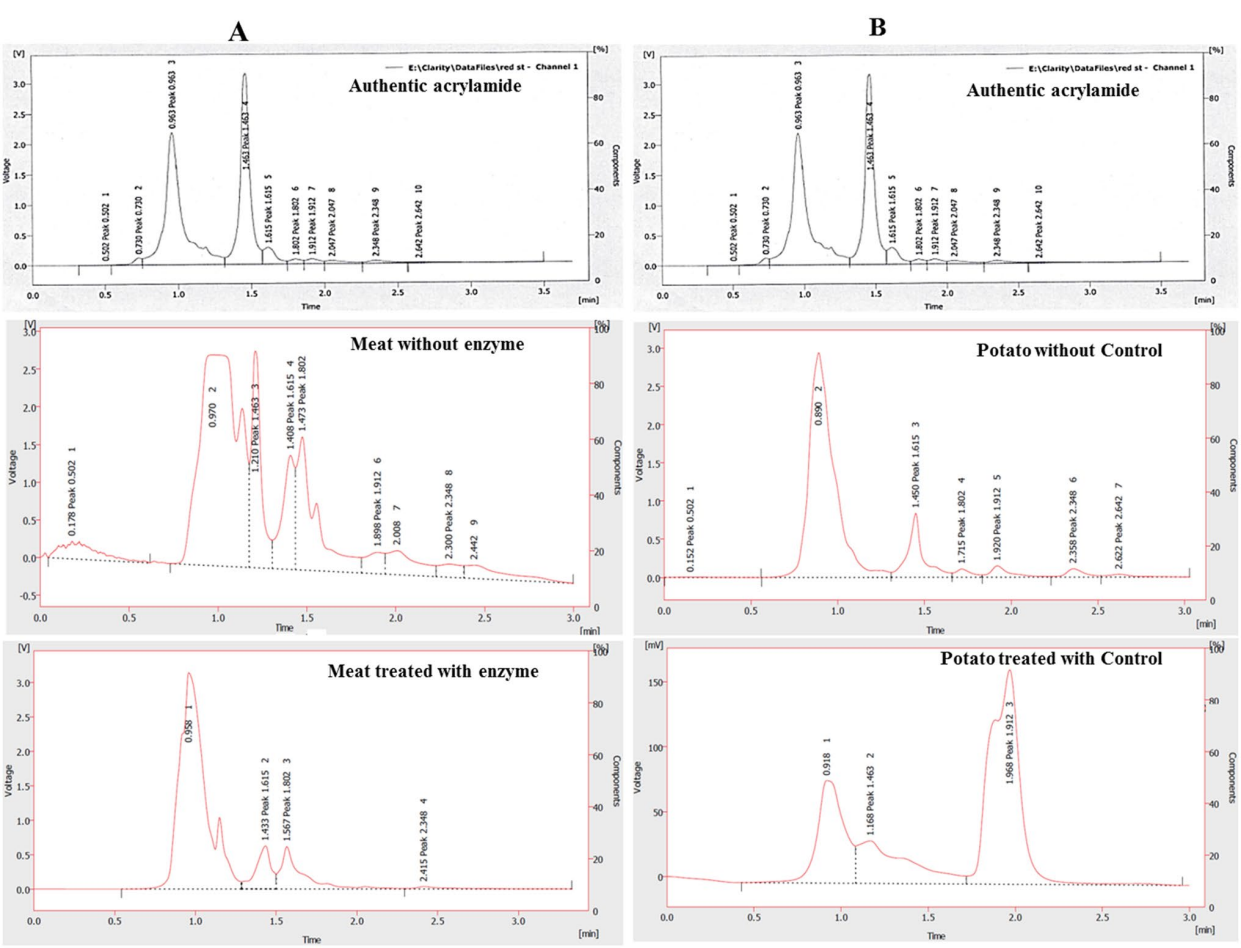
HPLC chromatogram for acrylamide determination on cooked meat (**a**) and potato chips (**b**) in presence and absence of the purified *A. fumigatus* acrylamide amidase. The fried meat and potato chips were soaked in water containing purified amidase for 2 h at 40 °C, comparing to control (without enzyme), and then pulverized in water prior to HPLC analysis. The acrylamide concentration was determined from the area under the peak area corresponding to the authentic acrylamide at 1.4-min retention time

**Fig. 3:**
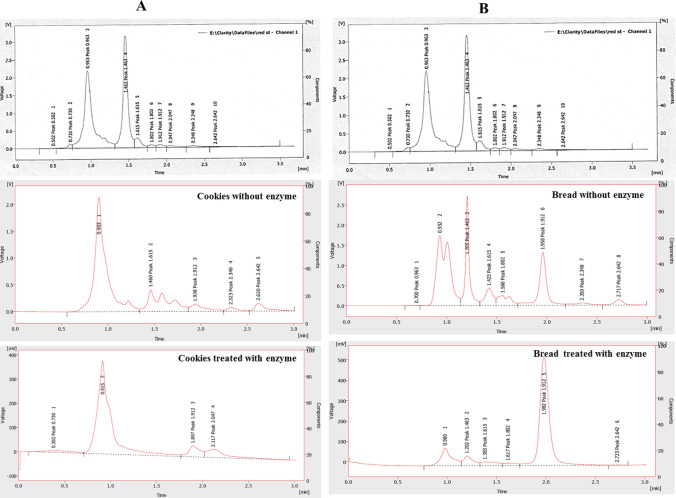
HPLC chromatogram for acrylamide determination on cookies (**a**) and bread (**b**) in presence and absence of the purified *A. fumigatus* acrylamide amidase. The cookies and bread were soaked in water containing the purified amidase for 2 h at 40 °C, comparing to control (without enzyme), and then pulverized in water prior to acrylamide determination by HPLC. The acrylamide concentration was determined from the area under the peak corresponding to the authentic acrylamide at 1.4-min retention time

The activity of the purified *A. fumigatus* amidase in degradation of acrylamide in different food products, namely meat, bread, cookies, and potato chips was estimated by HPLC. One gram of each food products was soaked in amidase preparation, incubated for 1 h at 40 °C, and then the acrylamide was extracted and their concentration was assessed. From the HPLC chromatograms (Figs. 2, 3), the acrylamide concentrations in the tested food products were extremely reduced upon implementation of amidase, comparing to control food products (without enzyme treatment). The concentration of acrylamide in the meat was reduced from 242.3 to 73.5 μg/g in response to the amidase, so, upon enzyme application the acrylamide amount was reduced by ~ 3.3 folds, ensuring the efficient functionality of enzyme in foods technology applications. While, acrylamide concentration of the tested cookies was dramatically reduced to 5.0 μg/g upon enzyme treatment comparing to control samples without enzyme treatment (104.4 μg/g), i.e., about 20 folds reduction to acrylamide (Fig. 3). Whereas, the initial concentrations of acrylamide in potato chips and bread were reduced from 90.0 and 45.2 μg/g to 6.0 and 4.0 μg/g, respectively, in response to the enzyme treatment (Fig. S3). The dramatic reduction of acrylamide concentration in the tested food products ensures the functionality of amidase in degradation of acrylamide into their corresponding by-products (ammonia and acrylic acids). The efficiency of amidase in degradation of acrylamide, as a post-acrylamide formation control, could be a co-supportive approach with L-asparaginase as a pre-acrylamide formation control. L-Asparaginase purified from *A. fumigatus* abolishes the acrylamide formation by degradation of asparagine, thus preventing the initial formation of acrylamide [18, 26, 47]. Similarly, the purified acrylamide-degrading enzyme “acrylamidase” from *S. acidaminiphila* MSU12 had a significant activity in degradation of authentic acrylamide into ammonia and acrylic acid [21, 48]. Determination of the amount of acrylamide and their degradation by-products “acrylic acid” by HPLC has been frequently recognized as an authenticated tool [9, 13, 15]. The probiotic *Lactobacillus acidophilus* has the ability to degrade acrylamide by acrylamide amidase [48]. Consistently, various bacterial species mainly *R. eutropha, X. maltophilia, Pseudomonas* sp., *E. aerogenes,* and *V. boronicumulans* have the ability to degrade acrylamide by production of acrylamide-degrading enzyme “acrylamidase” [4, 6, 12, 15, 48, 49]. Surprisingly, all the studies about acrylamide amidase “amidohydrolase” were reported only for authentic acrylamide degradation, but not in food applications in different food products, so, this is the first study evaluating the actual acrylamide-degrading potency by purified fungal amidase, in food products.

### LC–MS Analysis of Acrylamide Degradation By-Products in Response to Amidase in Foods

**Fig. 4:**
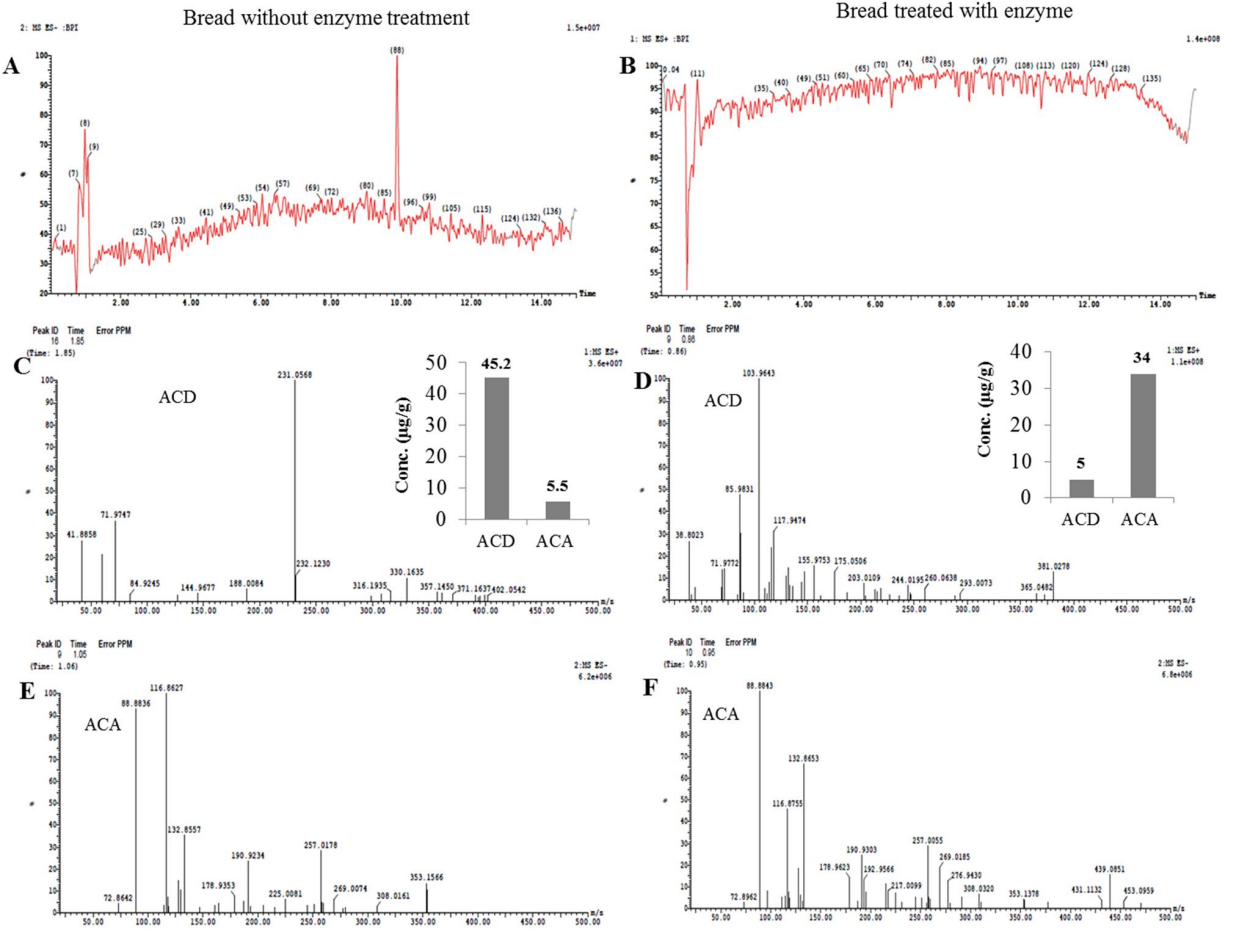
LC–MS analysis of acrylamide on bread treated with the purified *A. fumigatus* acrylamide amidase, comparing to enzyme-untreated bread. **a** Total ion current chromatograms (Counts vs. Acquisition Time (min)) of enzyme-untreated bread (**a**) and enzyme-treated bread (**b**). The mass spectra (*m/z*) of acrylamide (ACD) of molecular mass 71 *m/z* of enzyme-untreated bread with total area 1.82% (**c**) and enzyme-treated bread with total area 0.18% (**d**). The mass spectra (*m/z*) of acrylic acid (ACA) of molecular mass 72.06 *m/z* of enzyme-untreated (**e**) and enzyme-treated (**f**) bread

**Fig. 5:**
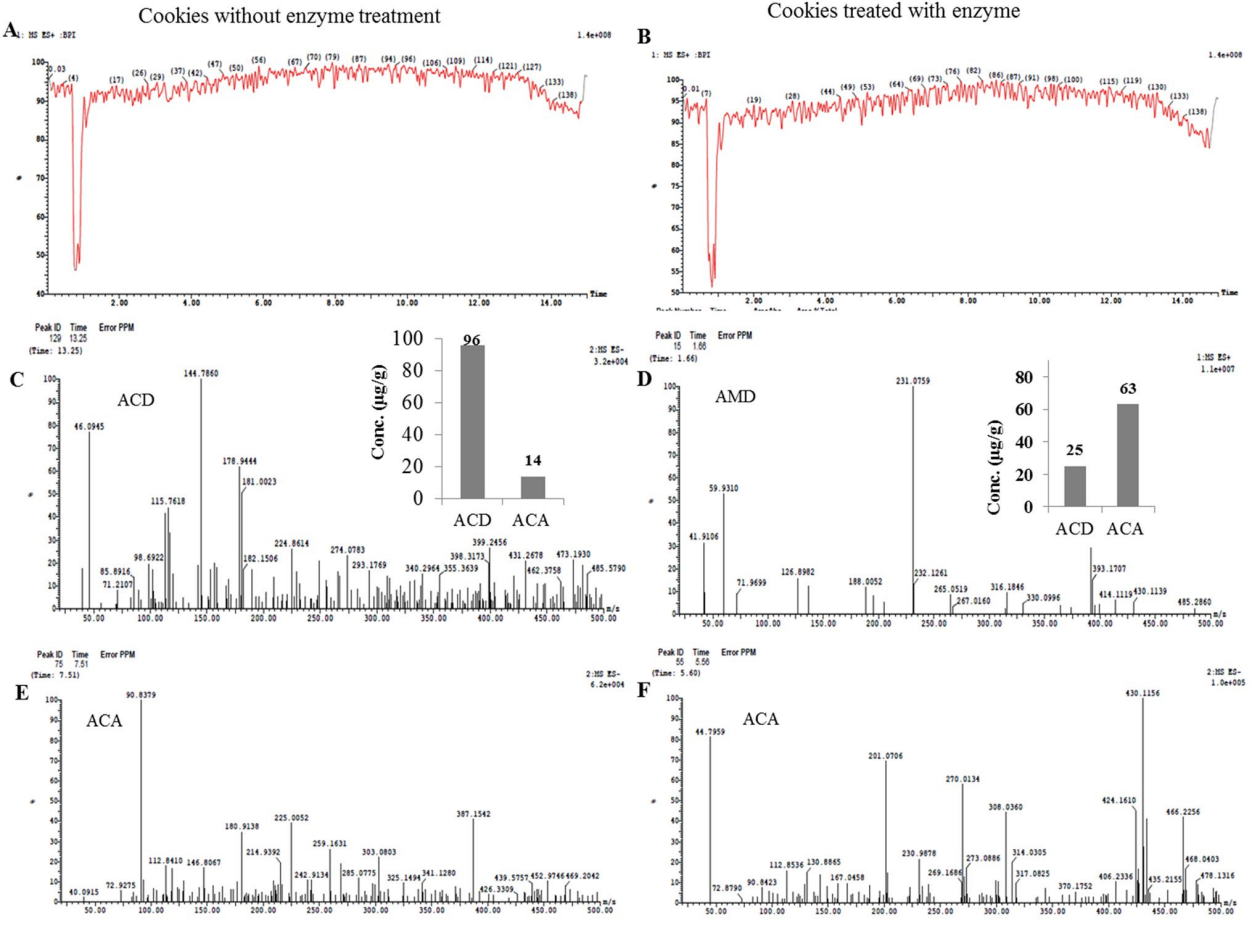
LC–MS analysis of acrylamide on cookies treated with the purified *A. fumigatus* acrylamide amidase, comparing to enzyme-untreated bread. **a**, Total ion current chromatograms (Counts vs. Acquisition Time (min)) of enzyme-untreated bread (**a**) and enzyme-treated cookies (**b**). The mass spectra (*m/z*) of acrylamide (ACD) of molecular mass 71 *m/z* of enzyme-untreated bread with total area 0.88% (**c**), and enzyme-treated cookies with total area 0.37% (**d**). The mass spectra (*m/z*) of acrylic acid (ACA) of molecular mass 72.06 *m/z* of enzyme-untreated (**e**) and enzyme-treated (**f**) cookies

The acrylamide degradation by-products in bread and cookies in response to treatment with *A. fumigatus* amidase were determined by LC–MS. After incubation of food products with the enzyme, the acrylamide and its by-products acrylic acid were extracted with hexane and their concentrations were determined by LC–MS. The LC–MS total ion current chromatogram and mass spectra of acrylamide and acrylic acid of bread samples treated with the enzyme comparing to control is illustrated in Fig. 5. From the spectral analysis, the amount of acrylamide with 71.06 m*/z* was decreased, while the acrylic acid with 72.06 m/z was substantially increased upon application of *A. fumigatus* amidase, ensuring the efficiency of enzyme for degradation of acrylamide into acrylic acid in food products. Upon treatment of bread with amidase, the amount of acrylamide was reduced to about 5.5 μg/g, comparing to 45.2 μg/g in the control sample (without enzyme treatment), i.e., by about nine-fold reduction to acrylamide upon enzyme treatment. However, the acrylic acid concentration of bread samples was increased to 34.0 μg/g in bread treated with enzyme, ensuring the enzyme functionality (Fig. 4).

The concentration of acrylamide and their degradation by-products “acrylic acid” in cookies in response to enzyme treatment was determined by LC–MS (Fig. 5). Practically, a noticeable degradation to the acrylamide and formation of acrylic acid in cookies in response to enzyme treatment was observed. From the results, the acrylamide concentration in cookies treated with enzyme was reduced to 25 μg/g, compared to 96.1 μg/g in control samples (without enzyme treatment). Sequentially, the acrylic acid concentration was increased from 14 μg/g to 43.3 μg/g in cookies, upon amidase treatment, ensuring the food efficiency of enzyme for acrylamide degradation. Similar results were reported for bioconversion of authentic acrylamide into acrylic acid upon treatment with acrylamidase from *S. acidaminiphila,* as revealed from the MALDI-TOF analyses [21, 49]. From the current results, the applicability of purified enzyme in the food degradation of acrylamide seems to be more affordable to avoid any toxic metabolites that might be released from the microorganisms during the processing. Since, all the previous studies on acrylamide degradation have been reported on authentic acrylamide and using natural fermentation by microorganisms, so, the current purified *A. fumigatus* amidase was the first report emphasizing the potency of this enzyme in the food applications, negating the possibility of toxic metabolites that might be released from the using of native microorganisms.

## Discussion

Acrylamide is one of the most hazardous by-products of Maillard reactions in foods during the processing, due to the reactivity of L-asparagine with the reducing sugars [1], especially in meat products, crisp bread, and French fries [2]. The acrylamide toxicity is usually related to neurotoxicity and carcinogenicity [3], due to their adduct formation with hemoglobin [7] and higher reactivity of epoxy glycidamide “by-products of acrylamide degradation” to form DNA adducts (glycidamide-DNA). The significant toxicity of acrylamide elaborates from its low molecular mass and higher water solubility, easily passing via the cellular biological membranes [11], with subsequent ability to undergo various metabolic transformations. Thus, preventing the acrylamide formation with L-asparaginase addition prior food processing is one of the most employed approaches for alleviation of acrylamide formation; however, efficiency of these methods is usually hampered by the presence of extra amounts of L-asparagine, leading to formation of acrylamide in foods [18]. So, degradation of “already-formed” acrylamide in foods could be the alternative affordable approach for complete abolishing of acrylamide toxicity. Thus, characterization of acrylamide-hydrolyzing enzyme “acrylamidase” from fungi with affordable thermal structural stability, catalytic efficiency, and evaluating their ability of acrylamide degradation in food products is the main objective.

*Aspergillus fumigatus*, *A. flavus*, *A. niger,* and *A. awomari* had the highest growth on PDA medium with acrylamide. A visual reduction to the growth of these fungi on acrylamide in a concentration-dependent manner was observed. Unlike to the reduction on growth of *A. flavus*, *A. awomari,* and *A. niger* at 0.5% acrylamide, *A. fumigatus* had a noticeable tolerance of acrylamide toxicity, and the biomass of *A. fumigatus* was only reduced by ~ 5% comparing to control. Similarly, *A. oryzae*, *Penicillium citrinum,* and *A. terreus* [15] and *Cupriavidus oxalaticus* and *Rhodococcus* sp. [19] have the potency to hydrolyze acrylamide. *Aspergillus fumigatus* was grown on PDB medium with 0.5% acrylamide and then the intracellular crude proteins containing acrylamidase were extracted and then purified. The activity of acrylamidase was increased by 2.8 folds by gel-filtration and ion-exchange chromatography, and the purified enzyme gave a single band of molecular mass 50 kDa under denaturing PAGE. Consistently, the molecular subunit structure of acrylamidase purified from *Rhodococcus* sp., *Pseudomonas aeruginosa,* and *Brevibacterium* was about 43.0–44.5 kDa [15, 19]. The maximum activity of the purified acrylamidase from *A. fumigatus* was reported at 35–40 °C, with a noticeable reduction to the enzyme activity by about 20%, comparing to control (35 °C). The half-life time (*T*_*1/2*_) of the purified *A. fumigatus* acrylamidase was 250, 75.7, 29.9, and 16.3 h, at 4, 20, 30, and 40 °C, respectively. The thermal denaturation rate (*Kr*) of amidase at 4 and 40 °C was 0.002 × 10^–3^ and 0.499 × 10^–3^ min. A noticeable reduction to the enzyme activity was observed at highly acidic and alkaline pHs. Similar biochemical properties of acrylamidase were reported [19]. The activity of the enzyme was reduced by ~ 40% by EDTA, confirming the metalloproteinic identity of this enzyme. The activity of amidase was restored upon addition Ca^2+^, Cu^2+^, and Ba^2+^, followed by monovalent cations K^+^ and Na^+^ at 1 mM. The enzyme activity was reduced by ~ 30% in response to PMSF, MBTH, and DTNB, ensuring the implantation of surface thiols amino acids on the catalytic sites of enzyme [50]. The cytotoxicity of the crude proteins of *A. fumigatus* was assessed in the experimental mice, with no signs of toxicity, based on the tested biochemical and hematological parameters [18].

The activity of the purified *A. fumigatus* acrylamidase in acrylamide degradation in food products such as meat, bread, cookies, and potato chips was assessed. The acrylamide content in the tested food products was extremely reduced upon implementation of amidase, comparing to control. The acrylamide content in meat was reduced by 3.4 folds in response to the enzyme, ensuring the efficient functionality of enzyme in foods applications. The acrylamide content of cookies was dramatically reduced by about 20 folds, upon the enzymatic treatment. The dramatic reduction of acrylamide contents in the food products ensures the functionality of amidase in degradation of acrylamide into ammonia and acrylic acids that could be a co-supportive approach with L-asparaginase as a pre-acrylamide formation control. The purified acrylamidase from *S. acidaminiphila* had a significant activity in degradation of authentic acrylamide into ammonia and acrylic acid [9, 13, 15, 21, 22, 24]. The probiotic *Lactobacillus acidophilus* has the ability to produce acrylamidase [46, 47]. Consistently, *R. eutropha, X. maltophilia, Pseudomonas* sp., *E. aerogenes,* and *V. boronicumulans* were reported to be acrylamidase producers [4, 6, 12, 15, 16] [21, 24]. The acrylamide and its by-products “acrylic acid” were extracted with hexane and their concentrations were determined by LC–MS. The amount of acrylamide was decreased, while the acrylic acid was substantially increased upon application of *A. fumigatus* acrylamidase, ensuring the efficiency of enzyme in food applications. Similarly, bioconversion of authentic acrylamide into acrylic acid upon treatment with acrylamidase from *S. acidaminiphila,* was reported [21, 49]. The applicability of purified enzyme in food degradation of acrylamide seems to be more affordable to avoid any toxic metabolites released from the microorganisms during the processing. Since, all the previous studies on acrylamide degradation have been reported on authentic acrylamide, and so, this is the first report emphasizing the potency of *A. fumigatus* acrylamidase in food applications, negating the possibility of toxic metabolites that might be released from using of native microorganisms.

## Conclusion

Acrylamide amidase was purified from *A. fumigatus* EFBL to its molecular homogeneity by gel-filtration and ion-exchange chromatography and their overall biochemical properties were studied. The acrylamide-degrading activity of the purified enzyme has been assessed in various food products. The acrylamide contents of the processed foods such as meat, cookies, potato chips, and bread were strongly reduced by ~ 90% in response to the enzyme treatment, comparing to control food samples, as revealed from the HPLC analysis. Upon addition of the purified amidase to the tested food products, the acrylamide was completely degraded to acrylic acid as revealed from the LC/MS profile, ensuring the functionality of this enzyme in food applications. Interestingly, this is the first report exploring the potency of amidase to hydrolyze the acrylamide compound in food products, authenticating their efficiency, selectivity to the acrylamide compound, and resistance to the other chemicals compounds that might inhibit their activity in foods. However, further biochemical analyses are required to assess the affinity of this enzyme for selectively hydrolyze acrylamide in foods, without affecting amino acids, and beneficial compounds that might be stereochemically similar to acrylamide.

### Supplementary Information

Below is the link to the electronic supplementary material.

Supplementary file1 (PPTX 3649 kb)

## Data Availability

All data generated during this study are included in this published article and its supplementary information files.
