# Supplementary material for: Biochemical Characterization of Thermostable Acrylamide Amidohydrolase from Aspergillus fumigatus with Potential Activity for Acrylamide Degradation in Various Food Products
Source: Curr Microbiol. 2023 Dec 5;81(1):30. doi: 10.1007/s00284-023-03544-1 (PMC10698087; doi:10.1007/s00284-023-03544-1)
Supplement: Supplementary file 1 — Supplementary file1 (PPTX 3649 kb) [file 284_2023_3544_MOESM1_ESM.pptx]

## Slide 1
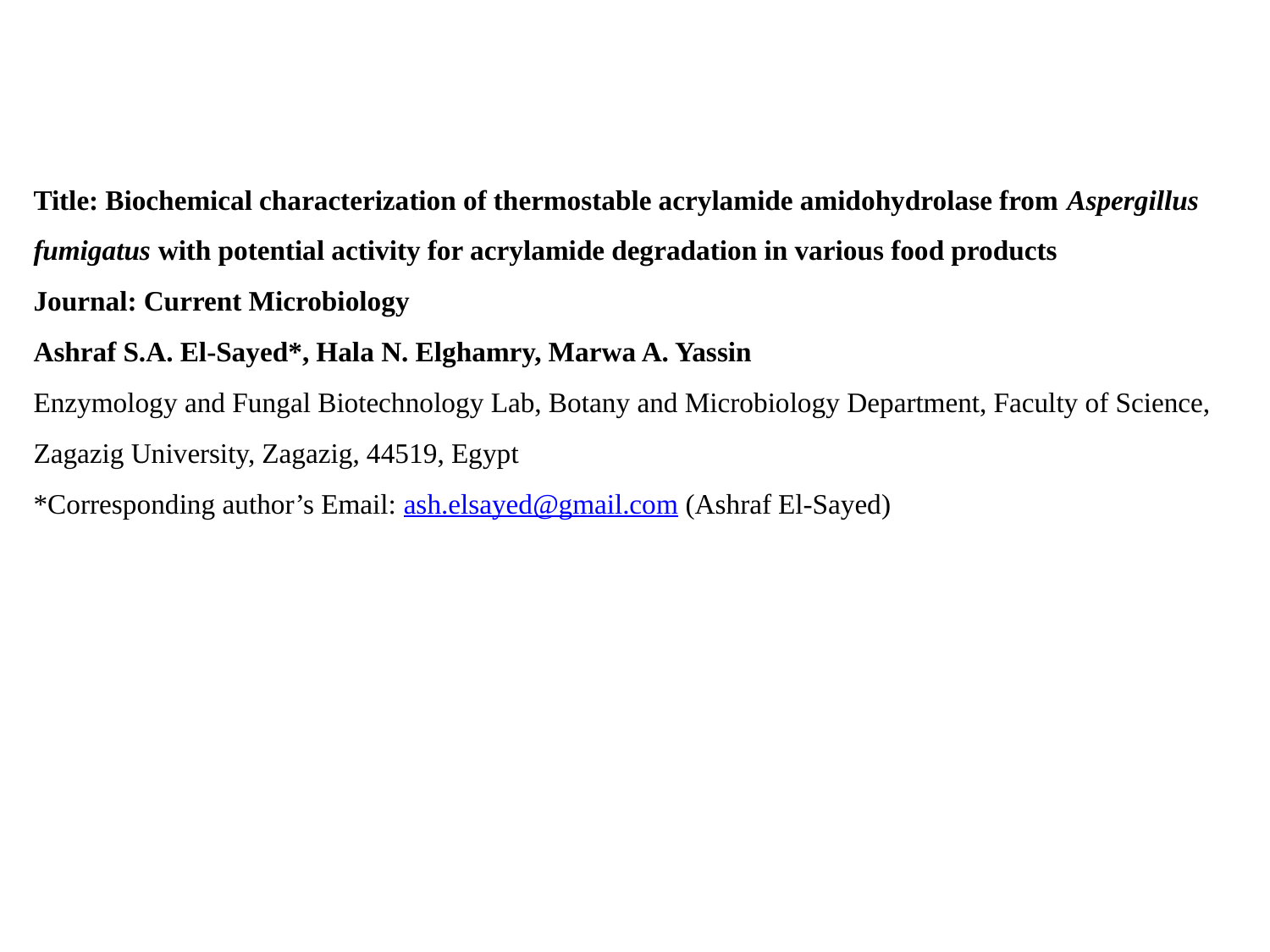

Title: Biochemical characterization of thermostable acrylamide amidohydrolase from Aspergillus fumigatus with potential activity for acrylamide degradation in various food products
Journal: Current Microbiology
Ashraf S.A. El-Sayed*, Hala N. Elghamry, Marwa A. Yassin
Enzymology and Fungal Biotechnology Lab, Botany and Microbiology Department, Faculty of Science, Zagazig University, Zagazig, 44519, Egypt
*Corresponding author’s Email: ash.elsayed@gmail.com (Ashraf El-Sayed)

## Slide 2
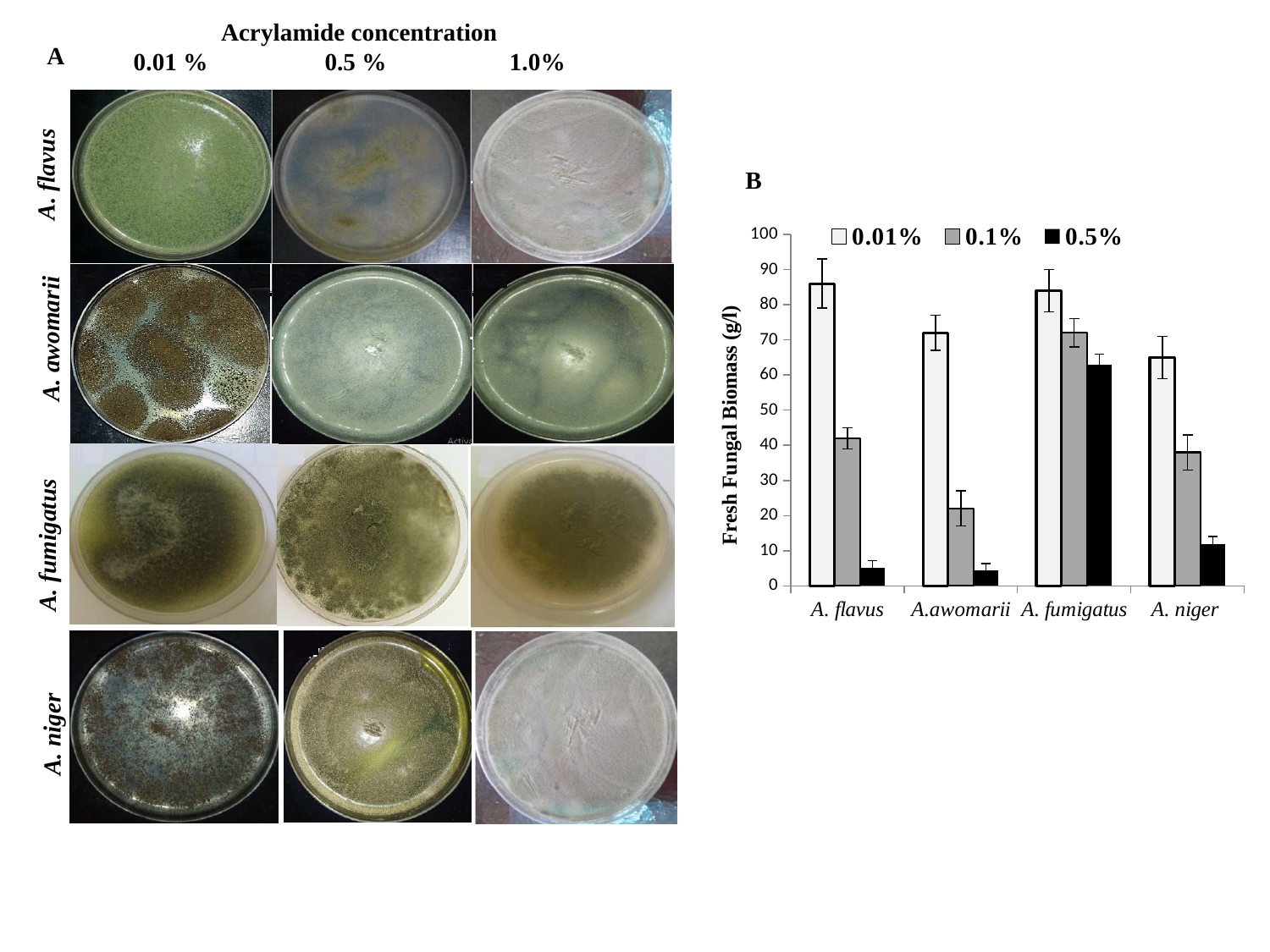

Acrylamide concentration
 0.01 % 0.5 % 1.0%
A. flavus
A. awomarii
A. fumigatus
A. niger
A
B
### Chart
| Category | 0.01% | 0.1% | 0.5% |
|---|---|---|---|
| A. flavus | 86.0 | 42.0 | 5.2 |
| A.awomarii | 72.0 | 22.0 | 4.3 |
| A. fumigatus | 84.0 | 72.0 | 63.0 |
| A. niger | 65.0 | 38.0 | 12.0 |

## Slide 3
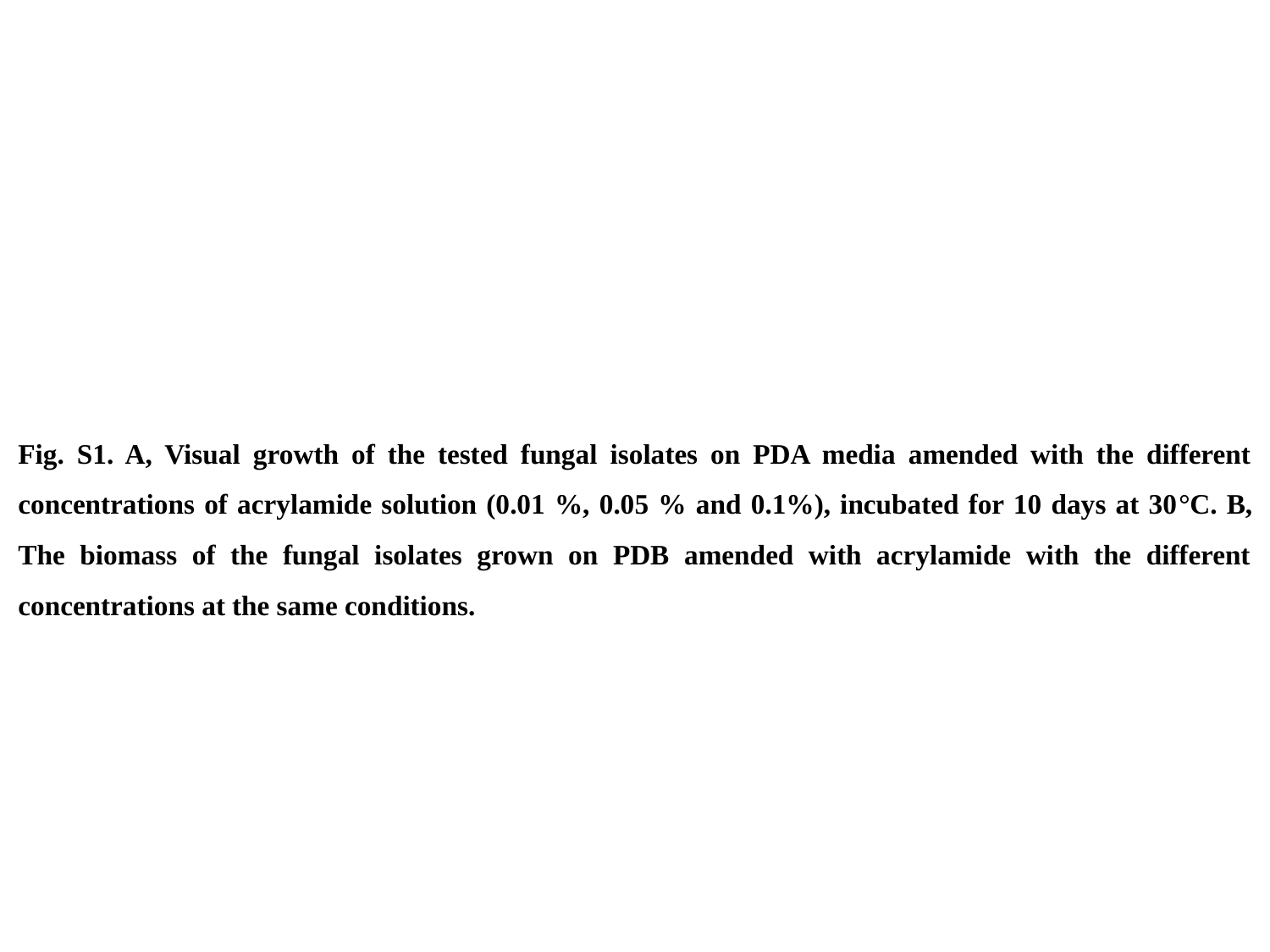

Fig. S1. A, Visual growth of the tested fungal isolates on PDA media amended with the different concentrations of acrylamide solution (0.01 %, 0.05 % and 0.1%), incubated for 10 days at 30°C. B, The biomass of the fungal isolates grown on PDB amended with acrylamide with the different concentrations at the same conditions.

## Slide 4
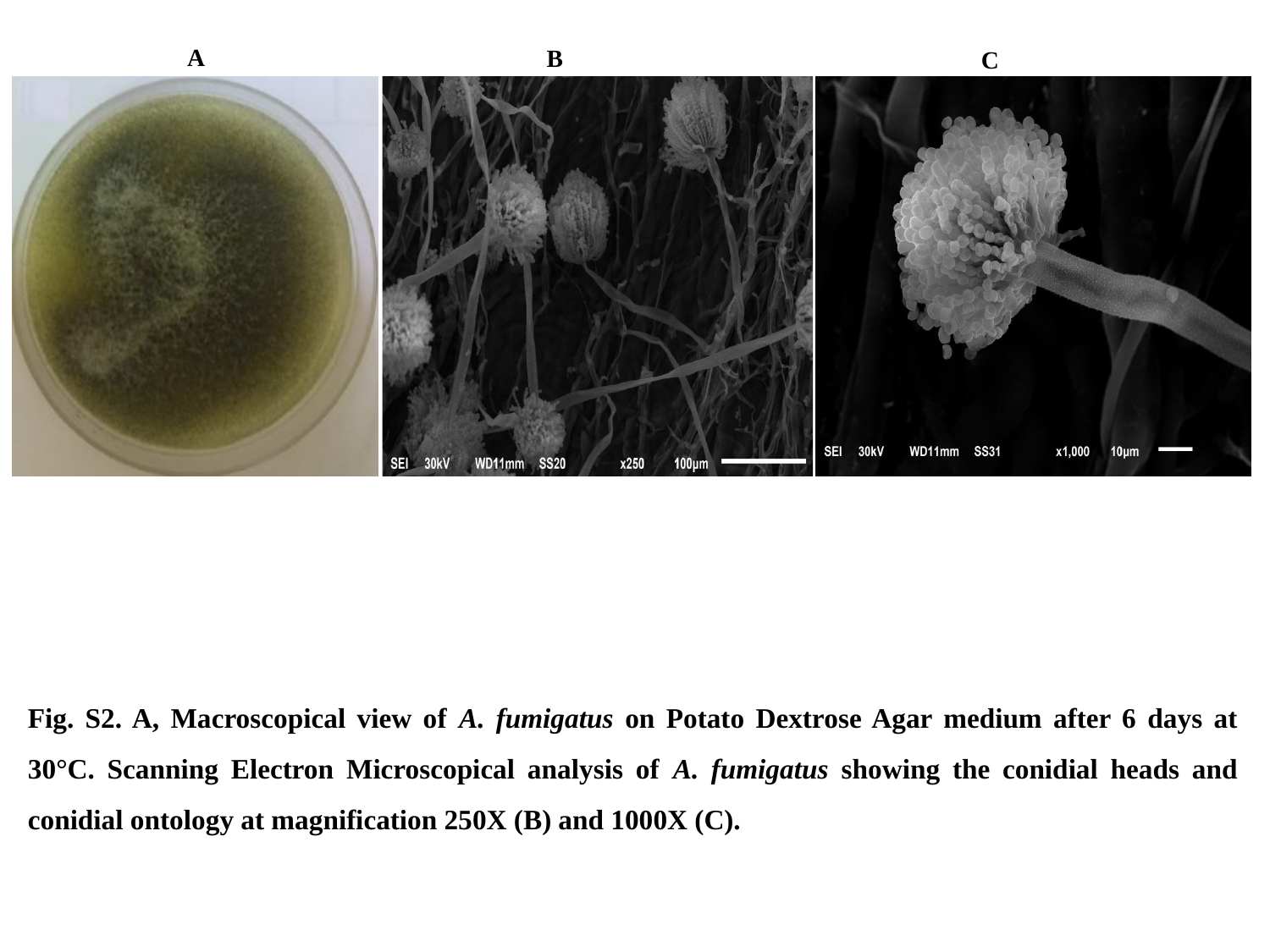

A
B
C
Fig. S2. A, Macroscopical view of A. fumigatus on Potato Dextrose Agar medium after 6 days at 30°C. Scanning Electron Microscopical analysis of A. fumigatus showing the conidial heads and conidial ontology at magnification 250X (B) and 1000X (C).

## Slide 5
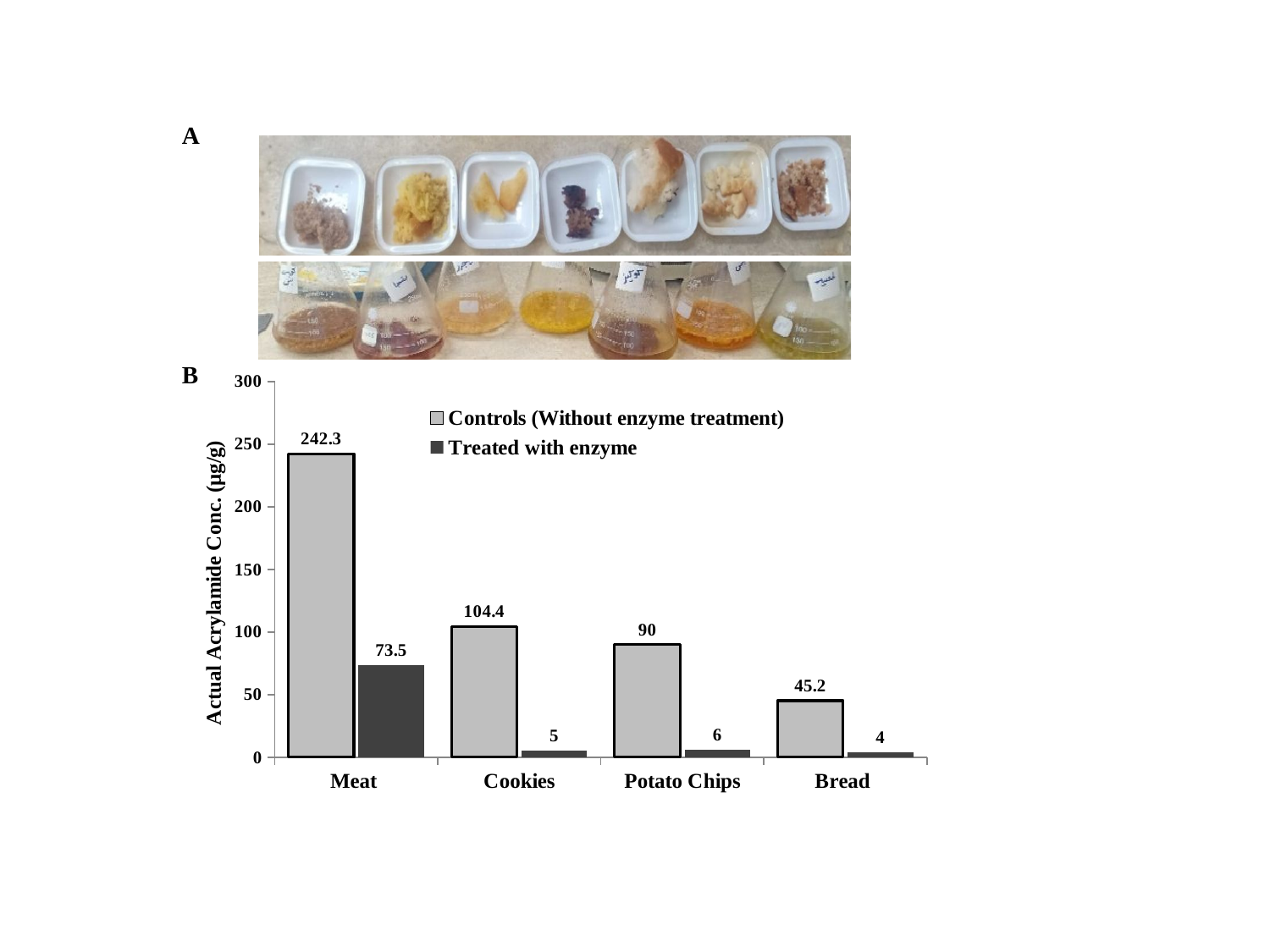

A
### Chart
| Category | Controls (Without enzyme treatment) | Treated with enzyme |
|---|---|---|
| Meat | 242.3 | 73.5 |
| Cookies | 104.4 | 5.0 |
| Potato Chips | 90.0 | 6.0 |
| Bread | 45.2 | 4.0 |B

## Slide 6
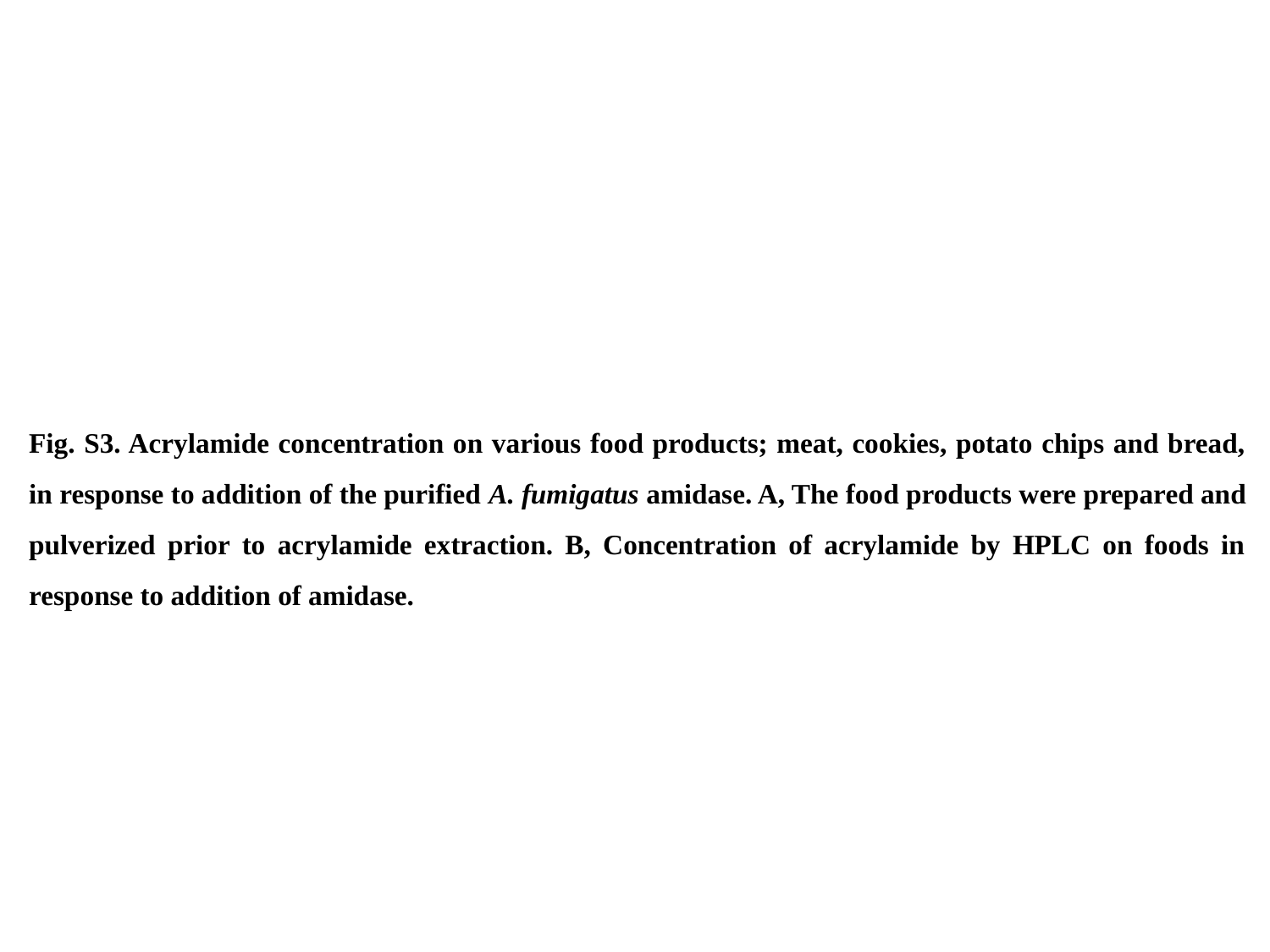

Fig. S3. Acrylamide concentration on various food products; meat, cookies, potato chips and bread, in response to addition of the purified A. fumigatus amidase. A, The food products were prepared and pulverized prior to acrylamide extraction. B, Concentration of acrylamide by HPLC on foods in response to addition of amidase.
